# Report on the Forest Trees Workshop at the Plant and Animal Genome Conference

**DOI:** 10.1002/cfg.262

**Published:** 2003-04

**Authors:** Christophe Plomion, Janice Cooke, Tom Richardson, John Mackay, Gerald Tuskan

**Affiliations:** 1 UMR BioGeco INRA 69 Route d'Arcachon Cestas Cédex 33612 France; 2 Centre de Recherche en Biologie Forestière Université Laval Québec Québec G1K 7P4 Canada; 3 New Zealand Forest Research Rotorua Private Bag 3020 New Zealand; 4 Oak Ridge National Laboratory Environmental Sciences Division Oak Ridge TN 37831-6422 USA


**Comparative and Functional Genomics**
*Comp Funct Genom* 2003; **4**: 229–238.
Published online 1 April 2003 in Wiley InterScience (www.interscience.wiley.com). **DOI:** 10.1002/cfg.262

**Conference Review**

### Report on the Forest Trees Workshop at the Plant and Animal Genome Conference

Christophe Plomion[1]*, Janice Cooke[2], Tom Richardson[3], John Mackay[2] and Gerald Tuskan[4]

[1] *UMR BioGeco, INRA 69 Route d'Arcachon, 33612 Cestas Cédex, France*
[2] *Centre de Recherche en Biologie Forestière, Université Laval, Québec, Québec, Canada G1K 7P4*
[3] *New Zealand Forest Research, Private Bag 3020, Rotorua, New Zealand*
[4] *Oak Ridge National Laboratory, Environmental Sciences Division, Oak Ridge, TN 37831-6422, USA*

**Correspondence to:*
*Christophe Plomion, INRA, 69 Route d'Arcachon, 33612, Cestas Cédex, France.*
*E-mail: plomion@pierroton.inra.fr*

Received: 25 January 2003
Accepted: 3 February 2003

### Introduction

Each week, progress is made in sequencing the genomes of species ranging from bacteria to humans. The genetic sequences of plant species such as *Arabidopsis* and rice have already been completed, and the first tree genome should be sequenced by 2003 (**http://www.ornl.gov/ipgc/**). Analysing this new information provides an unprecedented opportunity to understand the regulation of genes, the function of gene products and, eventually, how organisms are assembled. Just as genomics tools are acknowledged as a crucial component of strategies aimed at improving human health and the quality of life, we believe that genomic tools will be critical to ensure the long-term sustainability of forest health and productivity.

Since January 1993 — the date of the first Plant Genome Conference — the Forest Trees Genomics Community has held an annual workshop in San Diego (CA, USA). The Workshop has always provided an excellent forum for reviewing the current state of knowledge and discussing new directions of research. It has also provided opportunities for establishing contacts between scientists with different backgrounds (physiologists, molecular biologists, geneticists, bioinformaticians, biometricians), and new collaborations between participants. Since January 2002, the Workshop has also become the annual meeting of the 'Forest Genomics' working party of the International Union of Forestry Research Organizations (**http://iufro.boku.ac.at/iufro/iufronet/d2/hp20410.htm**). In 2003, this forum for presentation and discussion was divided into four sessions. The first session was devoted to genetic mapping and QTL detection. The second session focused on a quite new research field in the forest genomics community: SNP discovery and linkage disequilibrium mapping. Session three was devoted to functional genomics in conifer (mainly pines) and broadleaves (eucalypts and poplars) and session four was concerned by the International *Populus* Genome Consortium (IPGC). We will briefly summarize the workshop contributions.

### Session report

### Genetic mapping and QTL detection

This session emphasized the use of genetic linkage maps as essential tools to (a) provide information

Copyright © 2003 John Wiley & Sons, Ltd.

regarding the structure (**I. Scotti**) and evolution (**E. Hayahi, K. Krutovskii**) of conifer genomes, and (b) allow the detection of the loci (QTLs) that control quantitative variation, not only of agronomic traits (e.g. biomass, **S. Hanley**; wood quality, **D. Pot**; dormancy-related, **K. Jermstad**) but also of molecular traits (e.g. transcript accumulation, **M. Kirst**).

### Genome structure

As a step towards the understanding of the structure of the genome of conifers, **I. Scotti** (Udine University, Italy) presented an 'Analysis of the Distribution of Marker Classes in a Genetic Map of Norway Spruce'. He described a large-scale 'geography' of the genome of *Picea abies* through the application of statistical methods borrowed from geographical sciences (autocorrelation) that allowed the statistical testing of the clustering of molecular markers (AFLPs, ESTPs, S-SAPs, inter-LTRs, SSRs) of the same or different types (i.e. expressed or non-expressed regions, repetitive or low copy regions). This approach, applied for the first time to the analysis of the distribution of markers in a linkage map, produced an overview of the architecture of the genome of this species, suggesting that there is spatial autocorrelation among markers, i.e. similar markers appear to be clustered across the genome.

### Comparative mapping

Comparative mapping relies on the development and mapping of orthologous loci in related species, and comparing their location along linkage groups. The transfer of genetic information (e.g. markers, candidate genes, QTLs) between phylogenetically related species, and the study of genome evolution, are the most promising perspectives in developing comparative mapping studies. Genetic linkage maps of forest tree species have been widely developed in the last decade (reviewed by Cervera *et al.*, 2000), providing the framework for comparative mapping studies to expand over the past 3 years. The Conifer Comparative Genomics Project (CCGP, **http://dendrome.ucdavis.edu/Synteny/**) was the first project formed as an international collaboration to develop orthologous genetic markers. Expressed sequence tags (ESTs) were chosen for this study, given the low rate of transferability of microsatellite markers in conifers. ESTs cannot only be studied across different species, but they also represent functional genes. They can be developed relatively easily from existing cDNA/EST libraries and large gene discovery projects.

While the first comparative mapping studies only focused on *Pinus* species, **K. Krutovskii** (USDA Davis, USA) 'Comparative Mapping in Conifers Using EST Markers' demonstrated how EST polymorphisms (ESTPs) have helped to line up genetic maps between conifer genera (*Pinus, Picea, Pseudotsuga*). Overall, gene content (synteny) and order (co-linearity) were maintained, which agrees with other pairwise genome comparisons within the genus *Pinus* (reviewed in Chagne *et al.*, 2003) and supports the cytogenetic evidence that chromosome evolution in the family Pinaceae is conservative. He also reported on the progress of EST sequencing projects for Norway spruce (*Picea abies*) and Douglas-fir (*Pseudotsuga menziesii*), for which 4000 ESTs have been produced and compared to loblolly pine (*Pinus taeda*) ESTs. Homologies between the three EST sets were high.

As part of a breeding program aimed at developing elite trees resistant to pine wood nematode in *Pinus thunbergii*, **E. Hayahi** (Forest Tree Breeding Center, Japan) presented the 'Development of Cleaved Amplified Polymorphic Sequence Markers in Japanese Black Pine'. They used ESTPs to integrate multiple maps of Japanese black pine constructed with AFLP and RAPD markers. Out of 117 EST primer pairs reported in loblolly pine, 72 pairs amplified a single fragment for Japanese black pine. Single nucleotide polymorphisms were found in 15 EST fragments by sequence analysis. Restriction enzymes were then selected to detect polymorphism. Finally, these markers were placed on the linkage map of Japanese black pine.

### QTL detection

Most characters important to forestry, such as biomass production, wood quality, biotic and abiotic stress response are complex quantitative traits, resulting from a number of different genes interacting with each other and with the environment. These genes act together to provide a quantitative difference, and are referred to as quantitative trait loci, or QTLs. The availability of genetic maps in many forest tree species has made it possible to dissect quantitative traits into their Mendelian inherited components (the QTLs) and to improve our

Copyright © 2003 John Wiley & Sons, Ltd.

basic understanding regarding the genetic architecture of economically and ecologically important traits. Sewel and Neale (2000) recently reviewed the science of QTL mapping in forestry. As already described for other organisms (Mauricio, 2001), it appears from their meta-analysis that, while there are many genes underlying a quantitative trait in segregating populations, only a few of them account for a major proportion of the phenotypic variance, providing opportunities to increase breeding efficiency through their direct manipulation. In their review, Sewel and Neale (2000) concluded with the need for QTL verification and validation before a commitment to marker-aided selection can be made in tree breeding.

While QTL mapping experiments have often concerned a single pedigree in a given species, the alignment of genetic maps across species can be considered as a starting point for QTL validation. In 'Wood Quality Breeding in Maritime Pine: From Wood Properties Dissection to Marker-aided Selection', **D. Pot** (INRA, France) showed the usefulness of comparative mapping for verifying quantitative trait loci (QTLs) between species. The alignment of genetic maps between *Pinus taeda* and *Pinus pinaster* served to validate wood density and cell wall chemistry QTLs and to co-localize positional candidate genes controlling these traits. Coincidence of map positions supports the hypothesis that loci underlying natural quantitative variation have been conserved during long periods of evolutionary divergence. Three presentations showed how the accuracy of mapping QTLs can be achieved by increasing sample size (**S. Hanley**), analysing QTLs in multiple environments (**K. Jermstad**) or different genetic backgrounds (**D. Pot**).

**S. Hanley** (University of Bristol, UK), in 'Genetic Mapping of Important Agronomic Traits in Biomass Willows', described a program of research to provide markers linked to key agronomic traits related to biomass production for marker-assisted selections in European Willow (*Salix viminalis*). To improve the ability to locate QTL, two large-scale mapping populations of 950 full-sib individuals each were established, one being planted in two sites. Drawing information from the existing willow map, they genotyped the two progeny sets using 100 microsatellite markers. Preliminary trait mapping identified genomic regions putatively involved in all initial target traits (stem height and diameter, fresh and dry weights, rust resistance, beetle damage).

**K. Jermstad** (Institute of Forest Genetics, USA), in 'Mapping of Quantitative Trait Loci Controlling Adaptive Traits in Coastal Douglas Fir. III QTL by Environment Interactions and Verification', described an experiment revealing the underlying genetic factors influencing the adaptive capacity of Douglas fir. Using clonal replicates (20 ramets from 400 progeny) from a three-generation outbred pedigree, she estimated QTLs responding to environmental signals (winter chilling, spring temperature, moisture availability, photoperiod) or dormancy-related traits (bud set date, bud flush date, growth rhythm). Thirty-four QTLs were detected among all traits evaluated. The proportion of phenotypic variation explained by any individual QTL was 0.7–9.5%. Co-location of QTLs amongst growth cessation and rhythm traits was frequently observed. Few QTL $\times$ treatment interactions were found for growth initiation, whereas several were detected among the traits measured for growth cessation, many of which were repeatedly detected among traits. The repeated detection of QTLs for terminal bud flush was evaluated within and between mapping populations. QTLs for bud flush were estimated at two test sites and in one greenhouse trial, and compared with QTLs for bud flush that were previously identified in full-sib progeny from an earlier mating of the same parents. Repeated detection of these QTLs was observed on six linkage groups providing a strong case for the validation of these QTLs. In this pedigree, **K. Krutovskii** also reported on the co-location between candidate genes and the dormancy-related QTLs.

**D. Pot** presented a three-step strategy to provide the basic background to initiate a breeding program for wood and end-use properties in maritime pine. In a first step, classical quantitative genetics provided estimates of variability and heritability for a wide range of wood quality-related traits. In a second step, these traits were dissected into their QTLs, resulting in the identification of several genomic regions harbouring loci involved in trait variation. Comparative mapping with loblolly pine allowed for comparison of QTL locations between the two pine species. The position of two QTLs controlling wood density and cell wall components were found to be conserved between the two species. He also

Copyright © 2003 John Wiley & Sons, Ltd. 

showed how sequenced cDNAs from a tissue-specific library (i.e. tissues that express the desired trait, in this case wood forming tissue) can be identified and mapped. Potential positional candidate genes can then be identified in conjunction with QTL mapping. These approaches, however, did not reveal the actual genes underlying the QTLs. In a third step, a candidate gene approach was taken, starting with the identification of nucleotide polymorphisms in 20 genes thought to be important in determining wood properties. An association study in a diallel was conducted to evaluate the potential use of these markers in a wider genetic background to provide reliable early selection criteria for wood properties (see next session).

QTL detection of molecular traits (TQL and PQL for quantitative accumulation of transcript and protein, respectively) and the comparison with QTLs for agronomic traits may also be a powerful tool for identification of candidate genes (reviewed in Shields and O'Halloran, 2002). **M. Kirst** (NC State University, USA) spoke about 'Genetical Genomics of *Eucalyptus*: Combining Expression Profiling and Genetic Segregation Analysis'. He described an experiment that uses the genetic variation between related individuals in a segregating population and adds the analytical tools available for molecular markers to the analysis of genome-wide expression profile data. He obtained the transcript level profiles of 2658 genes for 91 full-sibs in a highly replicated micro-array experiment, and reported on the co-location between a major QTL for wood density and a TQL (transcript quantity locus) for one gene whose product is 89% identical to an *Arabidopsis* protein involved in regulation of cellular osmotic pressure. This gene was further mapped within the TQL peak region, indicating that it was probably involved in the control of its expression. Variation at this locus accounts for 25% of the variation in wood density in this family.

**U. Reyes-Valdes** (University A Narro, Mexico) spoke about 'Founder-origin informativeness in outbred pedigrees'. He used the Shannon entropy to measure informativeness of genetic maps and mapping populations for QTL analysis. Using a simulation, he showed that this method was computationally faster than the classical least squares approach and produced the same conclusions.

### SNP discovery, linkage disequilibrium and association mapping

Since its inception more than a decade ago, forest tree molecular genetic research has traversed the twin tracks of (a) genetic linkage analysis driven by DNA-based polymorphisms and (b) the hunt for major effect candidate genes controlling target traits, without much opportunity to pull the tracks together. Applications arising from the first track have, until recently, relied on detecting neutral marker-trait associations and implementing MAS (marker-assisted selection) to enhance genetic gain. In forest trees these marker–trait associations are usually pedigree-specific, owing to species-level, or pedigree-level, linkage equilibrium between the neutral marker and the causative genetic variant(s) underpinning trait variation. Such pedigree specificity is a major disadvantage in most forest tree improvement programs because, in practice, many families are deployed in production forests (e.g. due to multiplication bottlenecks and plantation-wide genetic diversity targets). The second track, leveraging major effect genes to alter traits of interest, has also not led to paradigm shifts in genetic gain because few genes with major effects on commercially important, quantitatively inherited traits have been discovered. This is true despite experimental populations and approaches capable of doing so. Moreover, where major effect genes have been discovered, there is not always a straightforward response from gene modification where, for example, pathway redundancy frequently obviates the alteration of one gene in biosynthetic pathways. However, as is often the case, there is 'innovation at the interface'! Recent technological advances in candidate gene discovery via transcript profiling, and latterly SNP discovery, have created the opportunity to bring these two tracks together by identifying and subsequently using polymorphisms that are in strong linkage disequilibrium (LD) with traits (Flint and Mott, 2001). This is the increasingly familiar 'association mapping' common in human genetics. If validated, these approaches hold tremendous potential in forest tree selection programs. Last year at this workshop we heard the preliminary plans to begin SNP validation experiments and this year three leading groups presented their results to date.

In 'Gene-assisted Selection (GAS) — A New Paradigm for Selection in Forest Tree Species?'

Copyright © 2003 John Wiley & Sons, Ltd. 

**P. Wilcox** (Forest Research, New Zealand) reported on his team's efforts to validate SNP–wood characteristic linkage disequilibrium (LD) in *Pinus radiata*. They used CAGE transcript profiling to identify more than 200 putatively differential ESTs and real-time PCR to validate the differential transcript levels. To date, 48/60 ESTs thus tested have been RT–PCR-validated as differential, and eight of these 48 have been genetically mapped using large full-sib pedigrees and pre-existing framework maps. A further four genes showing homology to genes involved in xylogenesis in maritime pine (based on collaborative work involving INRA scientists) have also been mapped in the same pedigree. Promisingly, five of these $(8 + 4 =)$ 12 mapped ESTs have co-localized to QTLs for juvenile wood density, an important target trait. The New Zealand team is now evaluating two genes by genotyping all of the SNPs within their full-length cDNAs on an association population of 2000 unrelated genotypes. This is an onerous and potentially limiting step, but previous work has shown that each SNP must be evaluated for LD with the trait, since intra-genic LD is low for these genes. Nonetheless, if these results are positive, then the team may well reach their vision of 'gene-assisted-selection' — where superior trees are selected based on their DNA sequence and costly phenotypic testing is reduced (or eliminated).

**D. Pot**'s report (see the first session) also updated the meeting on his progress towards validating SNPs within wood property-related genes in *Pinus pinaster*. The candidate genes for this project arose from three sources: functional candidate genes selected because they are involved in lignin or cellulose biosynthesis; expressional candidate genes identified by their different expression patterns in developing xylem; and ESTs previously mapped as QTL in a *P. pinaster* QTL mapping pedigree. Within these candidate genes, the team detected SNPs at rates ranging from 12.5/exon kb to 40/ intron kb. Association tests are well advanced for 12 candidate genes representing 50 SNPs, using a half-diallel population comprised of 12 parents (therefore 60 families). In contrast to the unrelated population approach described above, this approach tested LD between SNPs and traits by correlating the expected SNP genotype frequency in each family with the family's mean phenotypic data. To date, 11 of the 12 candidate genes tested thus far showed significant LD between SNPs and traits. Amongst these were associations between lignification genes and wood density or growth with SNPs accounting for up to 10% to trait variability. Clearly, validated effects of this magnitude will change the game in forestry genetics.

In 'Nucleotide Diversity in Loblolly Pine', **G. Brown** (University of California, Davis, CA, USA) updated the session on SNP diversity, LD and association tests in *P. taeda*. He reported that SNP frequency in *P. taeda* ranged from 1/196 bp for non-synonymous substitutions in exons to 1/37 bp in non-coding regions. Estimates of average nucleotide diversity were higher than found in humans but lower than in *Drosophila* and maize. Nonetheless, there is plenty of sequence variation to work with. Results for 18 genes confirmed findings in other pines showing that intragenic LD clearly exists and is highly variable. Intergenic LD evaluated across two approximately 20 cM intervals, each harbouring three genes, was negligible. If this is characteristic of pines, this suggests that whole-genome SNP scans to detect QTN will be inefficient. The team is now performing association tests. They are using a replicated population of 435 unrelated individuals, with two copies/genotype. Early results from 15 SNPs drawn from four candidate genes and evaluated against eight wood properties have revealed 'tantalizing suggestions' that the group plans to validate on more powerful association populations.

In the session's final talk, **G. Gill** (University of California, Davis, USA), in 'Detection of the Sequence Mutation Responsible for a Null Allele of Cinnamyl Alcohol Dehydrogenase in Loblolly Pine', reported on progress towards elucidating the nucleotide variants responsible for the most famous and best-characterized major gene in pine: the CAD null mutant in *P. taeda* (well described by MacKay *et al*., 1997). The homozygous CAD null mutant has CAD activity less than 1% of wild-type. A result of this CAD downregulation is a significant increase in stem growth, possibly owing to reallocation of resources from monolignol production (this relationship has been suggested in transgenic poplar). Sequencing the entire coding region from the wild-type and mutant allele revealed a tandem adenosine insertion in the null allele that creates a frameshift and leads to a premature stop codon. This sequence mutation is consistent with the phenotype. For routine genotyping of the sequence mutation, Gill and his team used the Perkin-Elmer 'acycloprime fluorescence polarization' methodology to develop an efficient SNP-based allele-specific assay. Trial testing of the assay on 160 *P. taeda* clones from nine different crosses that harboured the null allele accurately determined all of the genotypes; 400 plants were genotyped to estimate the frequency of the null allele and only two offspring were identified from the heterozgygous tree from which the original null allele was discovered. This result further supports the notion that the CAD null mutation is rare.

### Functional genomics

The presentations given in this session clearly demonstrated that functional genomics toolkits are now established in many forest tree research laboratories. The speakers highlighted the breadth of the toolkits in the forest tree functional genomic community, and emphasized that multiple approaches are often needed to dissect gene function. It was evident from these presentations that many forest tree functional genomics projects are using these tools to profile gene expression governing developmental or physiological processes that are more prominent in trees — such as wood formation — or that are a consequence of seasonal growth cycles associated with the perennial lifestyle of trees.

### Gene discovery and large-scale projects

ESTs and genomic sequences are the keystone to functional genomics. With these sequences in hand, it is possible to use a cornucopia of techniques to reconstruct the programs of gene expression that are involved in complex physiological and developmental phenomena. In a similar vein, it is also possible to determine the function of a previously uncharacterized gene shown to be associated with a specific process. The power of functional genomics comes from recently available large-scale and high-throughput technologies (Colebatch *et al.* 2002). These technologies enable the simultaneous analysis of hundreds or thousands of transcripts, proteins or metabolites; they also facilitate analysis of tens or hundreds of samples at the same time.

Two speakers (**J. Karlsson, S. Ralph**) presented large-scale projects that contain a significant EST sequencing component as part of their multi-faceted programs. Three other presentations (**U. Egertsdotter, S.-K. Yang, M. Stromvik**) highlighted projects associated with the large EST sequencing effort in loblolly pine headed by North Carolina State University (Allona *et al.*, 1998; Whetten *et al.*, 2001). Mention was also given to the *Populus* genome-sequencing project (**G. Tuskan**).

In 'The Swedish Poplar Genome Project', **J. Karlsson** (Plant Science Centre, Sweden) provided an update on the program now well under way at the Swedish Centre for Tree Functional Genomics (Sterky *et al.* 1998; Hertzberg *et al.*, 2001). This ambitious project brings together more than 20 PIs in an integrative, multidisciplinary program that includes EST sequencing, microarray transcriptome analysis, 2D PAGE/MALDI/QTOF proteome analysis, various kinds of mass spectrometry-based metabolite analyses, wood and fibre analysis, chemometrics, microscopy, bioinformatics, as well as a recently installed transgenic facility. As part of this latter capability, this group will undertake high-throughput analysis of gene knockouts created by RNAi technology, using both *Arabidopsis* and poplar. The group is investigating many aspects of poplar growth and development, as well as responses to the environment.

**S. Ralph** (University of British Columbia, Canada) presented 'Structural and Functional Approaches in Deciphering the Spruce and Poplar Genomes', a comparatively new large-scale project funded by Genome Canada. The overall structure and goals of the project on comparative genomics in poplar/aspen, spruce, and *Arabidopsis* were outlined, which include transcriptomics, proteomics and mapping. The development of full-length and normalized libraries for spruce EST sequencing was highlighted. Sequences arising from the spruce EST sequencing effort have now been used to construct first-generation microarrays. An overview of BAC end sequencing in poplar, which will be used to support the *Populus* genome sequencing project, was also described. In addition, progress on the development of *Arabidopsis arrays* with spotted long oligomers (70 nucleotides) was reported.

### Comparative genomics

The use of genomic sequence as a tool for comparative genomics was the focus of **G. Tuskan's**

Copyright © 2003 John Wiley & Sons, Ltd. 

(Oak Ridge National Laboratory, USA) presentation, 'The *Populus* Chloroplast Genome: A Comparison of Genome Structure and Organization'. This project has arisen from the *Populus* genome sequencing project. Chloroplast contamination in the DNA template used for sequencing has resulted in up to 450× coverage of the *Populus* chloroplast genome. Tuskan's group has used this finished genome to carry out comparative genomics studies, including investigations of phylogenetic relationships. They found that the gene order, gene number and genome size in the *Populus* chloroplast genome is highly conserved when compared to other plant genomes. The number of mutations fixed in non-synonymous positions appeared to be high relative to corn, lotus, rice and *Arabidopsis*, but is likely an artifact of the hypercorrect nature of the *Populus* sequence. Finally, this unprecedented sequencing depth has revealed intraclonal variation in chloroplast sequence that is the result of either heteroplasmy or chimerism.

### Bioinformatics

Processing, archiving and analysing data relating to thousands of genes at the same time demands approaches to data manipulation, annotation and others that have not been previously encountered in molecular biology (Tseng *et al*., 2001). Although many presentations in this session incorporated various aspects of bioinformatics, 'Bioinformatics of Loblolly Pine (*Pinus taeda*): An Overview of EST Data Resources' by **M. Stromvik** (University of Minnesota, USA) was dedicated to this vital component of functional genomics. This presentation covered the approaches that this group has used to archive, compile and annotate the loblolly pine EST project headed by North Carolina State University. Recent developments that will help to enable functional analysis include the implementation of metabolic reconstruction tools (ERGO Plants) and a copy of the Stanford Microarray Database. Both are implemented at the Center for Computational Genomics and Bioinformatics (**http://pine.ccgb.umn.edu/**).

### Expression profiling

The majority of talks in this session on functional genomics were devoted to expression profiling. Three presentations focused on gene expression associated with wood formation and wood properties. Many wood properties of interest to the forest products industry, including wood specific gravity, chemical composition and others, vary considerably between different parts of the tree, among trees of different species and, to a lesser degree, within species. It is generally assumed that variability in wood structure and reactivity is the result of adaptation and variability in the underlying process of wood formation. Transcript profiling studies are now being conducted in different groups to test different hypotheses linked to this assumption and to attempt to link gene expression, wood formation and wood properties. Several studies have implicated cell wall structural proteins or enzymes as differentially expressed in correlation with variation or changes in wood properties (reviewed in Plomion *et al*., 2001). Gene expression profiling of much broader arrays of genes are now becoming the focus of these studies, aimed at developing a more comprehensive understanding of the molecular events that unfold during secondary xylem differentiation in trees.

**U. Egertsdotter** (Institute of Paper Science and Technology, Atlanta, USA) presented 'Transcript Profiling in Wood Formation: Seasonal Variation and Induction of Compression Wood', linking microarray analyses of gene expression profiles with morphological analyses during wood formation over the course of a growing season and during the formation of compression wood induced naturally or by bending trees. This study has used a mixed linear model for statistical analysis of microarray data and revealed significant differential expression, even with differences as small as 1.4-fold, given appropriate replication of hybridized cDNAs and slides. This analysis showed that significant differential gene expression exists between the earlywood and latewood formation phases and that expression appears coordinated among genes encoding enzymes of related function or belonging to the same pathway, e.g. many genes belonging to the lignin biosynthesis pathway had higher transcript levels during the latewood formation.

**G. Le Provost** (INRA, France) presented 'Wood Formation in Maritime Pine by Transcriptome and Proteome Analysis', a study that is using cDNA–AFLP and 2D gel electrophoresis to compare earlywood vs. latewood, and reaction wood vs. opposite wood. This study explored changes that

Copyright © 2003 John Wiley & Sons, Ltd. 

can occur within a single tree as a result of the seasonal growth cycle and mechanical stress on wood cells which occurs when a tree is bent (compression wood). It identified changes in xylem transcript profiles during the induction of compression wood, and showed that the response varies significantly at different times during the active growth season. Both transcriptome and proteome analysis on the same samples showed greater differences between earlywood vs. latewood compared to compression wood vs. opposite wood.

**S.-K. Yang *et al.*** (Texas A&M, USA) presented 'Gene Expression in Earlywood and Latewood of Loblolly Pine from Different Geographical Regions', reporting on the use of microarray analyses and quantitative PCR to compare gene expression profiles during early and latewood formation, as well as wood formation of trees from two provenances in the USA that display markedly different wood density. Their results to date reveal relatively few differences in gene expression between both provenances, although comparatively more differences were found during the latewood rather than earlywood growth phase.

Three other presentations focused on forest tree responses to the environment. Studies such as these provide important insights into how trees respond to environmental cues at the molecular level, and how these responses impact economically important traits, such as growth rates, wood quality and cold hardiness. These studies also have the potential to address ecological issues. As integral components of natural forest ecosystems, forest trees present what is perhaps a unique gateway into the domain of environmental genomics. Each of the speakers presented projects that are combining physiological analyses with gene expression profiling to examine topics relevant to climate change (more generally referred to as global environmental change): adaptation to temperature, elevated $CO_2$ and terrestrial nitrogen availability (Sala *et al.*, 2000; Cooke *et al.*, 2003).

In 'Coldtree: Unraveling Molecular Events Underlying Dormancy and Cold Hardiness in Forest Tree Seedlings', **M. Van Wordragen** (ATO, The Netherlands) described a project using microarrays to find changes of gene expression associated with specific physiological stages of dormancy and cold hardiness in beech and Scots pine. This initial analysis revealed that dehydrins appear to be important in these responses.

**G. Taylor** (University of Southampton, UK) presented 'Global Gene Expression of a Poplar Forest Following Long-term Adaptation to Future $CO_2$ Concentrations', a project that is a part of the POPFACE experiments in Italy. Taylor's group has used microarrays to define groups of genes that are upregulated or downregulated in poplars in response to elevated levels of carbon dioxide. Analysis of these data suggests processes that may be involved in this response, including ethylene biosynthesis.

And in the presentation 'Using a Functional Genomics Approach to Delineate Carbon–Nitrogen Dynamics in *Populus*', **J. Cooke** (Université Laval, Canada) outlined a project combining differential display, macroarray analysis, manipulative physiology, biochemical analyses and transgenic plants to examine resource allocation and partitioning in poplar. Nitrogen-responsive gene expression was correlated with concomitant changes in growth and architecture, including wood properties.

### The International Populus Genome Consortium

The first official meeting of the International *Populus* Genome Consortium (IPGC), chaired by **G. Tuskan**, was held during the PAG XI meeting. As a result of the forest genetics community's efforts over the past decade to build a strong justification for why trees should be viewed as model systems in plant biology, and the US Department of Energy (DOE), Office of Science's decision to sequence the first forest tree genome — *Populus* — the IPGC was founded.

Sequencing the genome of a forest tree will undoubtedly usher in an exciting period of scientific discovery for the forestry community. The US DOE's Joint Genome Institute, with funding through the Office of Biological and Environmental Research, will provide a 3× draft sequence of the female black cottonwood (*P. trichocarpa*) clone 'Nisqually-1' in early 2003 and a second 3× draft in late 2003. Alignment of assembled contigs into chromosomal units will then be based on information from physical and genetic maps. It is anticipated that annotation will take place using *Populus*-specific gene finding models trained from full-length cDNA sequences provided by the Umeå Plant Science Center, Genome British Columbia, Oak Ridge National Laboratory and INRA. All

Copyright © 2003 John Wiley & Sons, Ltd.

genomic sequence data will be made freely available to the scientific community. Scientists worldwide will soon have an opportunity to examine fundamental mechanisms that determine growth and developmental processes in long-lived organisms. However, this same opportunity will also challenge the forest biologist to consider how such a resource should best be developed and used.

As a means to advance the capabilities of the forest science community in areas of functional and comparative genomics, the IPGC was formed. Multinational academic, government and private groups, working together, will help ensure that the tools, techniques and resources required to enter a molecular era are available to the forest research community. The IPGC will seek to promote cottonwood, aspen and hybrid poplar as model organisms (Wullschleger *et al.*, 2002) and to foster the development of a collaborative worldwide network of groups interested in tree biology. One of the benefits associated with IPGC will be the coordinated assembly of institutions and scientists working on poplar genomics. It will be the goal of this group to extend and lead post-sequence research activities in poplar, and to develop a collaboratively written science plan for the benefit of the science community. An international *Populus* Science Plan will include a far-reaching summary of the applications and opportunities associated with the arrival of the *Populus* genome sequence. Science teams are now being formed to examine genetic and genomic resources currently available to *Populus* researchers, to identify areas in which tools, techniques and additional resources must be developed, and to assess applications and opportunities for future research. The IPGC has enlisted over 200 members from 29 countries to help in this endeavour. In general, the Science Plan will focus on technical areas identified as critical to the future success of functional and comparative genomics in *Populus*. Currently, teams are being formed to address the following areas: genetic resources, physical mapping and marker development, tissue culture and transformation, gene expression/microarrays, metabolic characterization and metabolomics, protein characterization and proteomics, high-throughput phenotyping, and informatics, annotation and database developments. A time-line for development and delivery of an International *Populus* Science Plan suggests that a final science plan will be available towards the end of 2003. Scientists from all disciplines interested in participating in the consortium or in helping to develop the science plan are invited to register on the IPGC website (**http://www.ornl.gov/ipgc**).

### Conclusions and future prospects

The Forest Tree Workshop, organized within the PAG XI meeting, was considered very successful by its participants. The workshop brought together scientists with different backgrounds and provided an excellent opportunity to review the different aspects of forest tree genomics. It clearly shows that the genomic (r)evolution is providing forest tree researchers with large amounts of data, providing enormous opportunities to study and understand the genetics of ecologically and economically important traits for trees. In practice, genomic information should enable scientists to gain new insights into the variability and expression of genetic sequences to better evaluate the inherent nature of our forest genetic resources, e.g. this knowledge should be useful in breeding programs to accelerate the efficiency of selection and to deliver improved varieties for abiotic and biotic stress adaptation, wood production and wood quality. It will also be germane to conservation programs, where information on the genetic control of the physiological functions of trees should make it possible to preserve critically important genotypes. Another important area of application for functional genomics in particular is the adaptation of trees to environmental changes of all sorts, and physiological responses to biotic and abiotic stresses.

Although we are beginning to understand the genetic architecture of quantitative traits, we still know very little of the actual loci responsible for quantitative variation. EST sequencing and expression analysis are discovering remarkable variation in gene sequence and gene expression, thus identifying many good candidate genes. Now the challenge is to discover whether such variations really matter and whether they affect phenotypic variation in natural populations. If we believe that such a challenge may be tackled through multidisciplinary cross-overs between scientists involved in tree genomics, we also believe that our community should work closely with physiologists, ecologists, wood technologists, entomologists, pathologists,

Copyright © 2003 John Wiley & Sons, Ltd.

etc. to define the right traits to analyse. The importance of multidisciplinary approaches becomes evident as we undertake linkage disequilibrium, or association studies, between DNA sequence polymorphisms at candidate gene loci and quantitative traits. When carried out in well-selected natural populations, such studies are considered to offer a highly promising avenue to identify molecular mechanisms contributing to phenotypic variation. Ultimately, they are expected to lead to the identification of quantitative trait nucleotides. However, given the abundance of candidate genes, the problem now lies in the screening of the most relevant candidates and in associating genetic variation with the right traits and phenotypes. In order to achieve this goal, we will need to broaden our expertise to identify the best candidate genes and the right candidate traits.

### References

Allona I, Quinn M, Shoop E, *et al*. 1998. Analysis of xylem formation in pine by cDNA sequencing. *Proc Natl Acad Sci USA* **95**: 9693–9698.

Cervera MT, Plomion C, Malpica C. 2000. Molecular markers and genome mapping in woody plants. In *Molecular Biology of Woody Plants*, vol 1, Jain SM, Minocha SC (eds). Kluwer Academic: Dordrecht; 375–394.

Chagné D, Brown G, Lalanne C, *et al*. 2003. Comparative genome and QTL mapping between maritime and loblolly pines. *Mol Breed* (in press).

Colebatch G, Trevaskis B, Udvardi M. 2002. Functional genomics: tools of the trade. *New Phytol* **153**: 27–36.

Cooke JEK, Brown KA, Wu R, Davis JM. 2003. Gene expression associated with N-induced shifts in resource allocation in poplar. *Plant Cell Environ* (in press).

Flint J, Mott R. 2001. Finding the molecular basis of quantitative traits: successes and pitfalls. *Nature Rev Genet* **2**: 437–445.

Hertzberg M, Aspeborg H, Schrader J, *et al*. 2001. A transcriptional roadmap to wood formation. *Proc Natl Acad Sci USA* **98**: 14732–14737.

MacKay JJ, O'Malley DM, Presnell T, *et al*. 1997. Inheritance, gene expression and lignin characterization in a mutant pine deficient in cinnamyl alcohol dehydrogenase. *Proc Natl Acad Sci USA* **94**: 8255–8260.

Mauricio R. 2001. Mapping quantitative trait loci in plants: uses and caveats for evolutionary biology. *Nature Rev Genet* **2**: 370–381.

Plomion C, Le Provost G, Stokes A. 2001. Wood formation in trees. *Plant Physiol* **127**: 1513–1523.

Sala OE, Chapin FS, Armesto JJ, *et al*. 2000. Global biodiversity scenarios for the year 2100. *Science* **287**: 1770–1774.

Sewel MM, Neale DB 2000. Mapping quantitative traits in forest trees. In *Molecular Biology of Woody Plants*, vol 1, Jain SM, Minocha SC. (eds). Kluwer Academic: Dordrecht; 407–424.

Shield DC, O'Halloran AM. 2002. Integrating genotyping data with transcriptomic and proteomic data. *Comp Funct Genom* **3**: 22–27.

Sterky F, Regan S, Karlsson J, *et al*. 1998. Gene discovery in the wood-forming tissues of poplar: analysis of 5692 expressed sequence tags. *Proc Natl Acad Sci USA* **95**: 13330–13335.

Tseng GC, Oh M-K, Rohlin L, Liao JC, Wong WH. 2001. Issues in cDNA microarray analysis: quality filtering, channel normalization, models of variation, and assessment of gene effects. *Nucleic Acid Res* **29**: 2549–2557.

Whetten R, Sun YH, Zhang Y, Sederoff R. 2001. Functional genomics and cell wall biosynthesis in loblolly pine. *Plant Mol Biol* **47**: 275–291.

Wullscleger SD, Jansson S, Taylor G. 2002. Genomics and forest biology: *Populus* emerges as the perennial favorite. *Plant Cell* **14**: 2651–2655.

Copyright © 2003 John Wiley & Sons, Ltd. 
